# Tezepelumab: redefining TSLP blockade in severe asthma through mechanistic precision and translational pharmacology

**DOI:** 10.3389/fphar.2026.1732906

**Published:** 2026-04-24

**Authors:** Ghaith K. Mansour, Santosh Kumar, Hatouf H. Sukkarieh

**Affiliations:** 1 Alfaisal University, Riyadh, Saudi Arabia; 2 Department of Pharmacology, College of Medicine, Alfaisal University, Riyadh, Saudi Arabia

**Keywords:** alarmins, biologic therapy, precision medicine, severe asthma, tezepelumab, thymic stromal lymphopoietin, TSLP blockade, type 2 inflammation

## Abstract

Approximately 40%–50% of patients prescribed GINA Step 4–5 ICS/LABA therapy remain inadequately controlled, and existing biologics are restricted by phenotypic eligibility criteria that exclude the substantial type 2-low patient population. Thymic stromal lymphopoietin (TSLP) is an epithelial-derived alarmin cytokine that functions as a key upstream orchestrator of both type 2 and non-type 2 inflammatory pathways in asthma pathogenesis. Tezepelumab, a human monoclonal antibody targeting TSLP, represents the first biologic approved for severe asthma without phenotype- or biomarker-restricted eligibility, including type 2-low disease, with the caveat that the magnitude of benefit is somewhat smaller in T2-low patients. This review provides a comprehensive analysis of tezepelumab’s molecular pharmacology, structural basis of target engagement, clinical pharmacokinetic/pharmacodynamic profile, pivotal clinical trial evidence, safety data, and regulatory positioning. We systematically evaluate tezepelumab’s mechanistic rationale, therapeutic efficacy, biomarker correlations, and real-world implementation considerations, including pharmacoeconomic and access barriers. Furthermore, we critically discuss the current limitations of clinical trial evidence, population selection biases, real-world applicability concerns, and the need for long-term outcome data. Future research directions encompassing predictive biomarker development, expansion into non-asthma indications, real-world evidence generation, and advanced mechanistic studies are outlined. Tezepelumab exemplifies precision respiratory medicine by targeting upstream inflammatory cascades and establishes a paradigm for next-generation asthma therapeutics.

## Graphical Abstract

**Figure:**
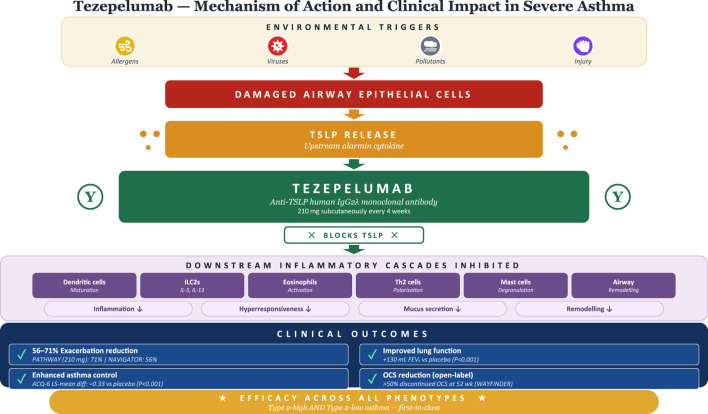

## 1 Introduction

Severe asthma represents a heterogeneous syndrome afflicting approximately 5%–10% of the global asthmatic population, imposing disproportionate morbidity, healthcare utilization, and diminished quality of life despite maximal inhaled corticosteroid and long-acting bronchodilator therapy (Côté et al., 2020; Rabe et al., 2023). Despite adherence to GINA Step 4–5 treatment, combining high-dose ICS with LABA and, where indicated, additional controllers, Multiple large registries and cohort studies indicate that around half of patients with severe asthma remain uncontrolled, with frequent exacerbations, persistent airflow limitation, and/or chronic systemic corticosteroid use despite high-intensity therapy (Chen et al., 2018). This failure of conventional pharmacotherapy is mechanistically rooted in molecular heterogeneity: steroid insensitivity is driven by overexpression of glucocorticoid receptor beta isoform (GRβ), which competitively antagonizes canonical GRα-mediated anti-inflammatory transcription, and by hyperactivation of phosphoinositide 3-kinase delta (PI3K-δ), which impairs histone deacetylase 2 (HDAC2) function and reduces corticosteroid responsiveness at the chromatin level (Chen et al., 2018). Neutrophilic and paucigranulocytic inflammatory endotypes, overrepresented in steroid-refractory disease, are largely unresponsive to ICS-mediated eosinophil suppression (Chen et al., 2018; Rönnebjerg et al., 2021; Reddel et al., 2021).

Traditional dichotomous classification into type 2-high versus type 2-low inflammation inadequately captures the complex, overlapping molecular endotypes that characterize this disorder (Hinks et al., 2021). The development of targeted biologic therapies has expanded the treatment armamentarium; however, each approved agent carries phenotypic eligibility restrictions that limit its applicability. Omalizumab (anti-IgE) requires documented perennial allergen sensitization and an IgE level within a defined range (30–700 IU/mL), excluding patients with non-allergic phenotypes. Mepolizumab and benralizumab (anti-IL-5/IL-5Rα) require blood eosinophil thresholds (≥150–300 cells/µL), conferring limited benefit in type 2-low patients who may account for 30%–50% of the severe asthma population (Chen et al., 2018). Dupilumab (anti-IL-4Rα) demonstrates its greatest efficacy in patients with elevated eosinophils or FeNO, with substantially attenuated benefit in type 2-low subgroups (Agache et al., 2020). This leaves a therapeutically underserved population—patients with recurrent exacerbations but low or absent type 2 biomarkers—without an evidence-based biologic option. The recognition that epithelial barrier dysfunction and alarmin release constitute upstream initiating events in asthma pathogenesis has catalyzed development of therapeutics targeting these proximal mediators (Pelaia et al., 2020; Lindsley et al., 2025).

Thymic stromal lymphopoietin (TSLP), a four-helix bundle cytokine belonging to the interleukin-2 family, is released by damaged airway epithelial cells following exposure to allergens, viruses, particulate matter, and mechanical injury (Gauvreau et al., 2020). Unlike downstream type 2 cytokines such as IL-5 and IL-13, TSLP functions as a upstream key orchestrator activating dendritic cells, group 2 innate lymphoid cells (ILC2s), T lymphocytes, mast cells, eosinophils, and airway structural cells (Roan et al., 2019). This pleiotropic activity positions TSLP as a rational therapeutic target for severe asthma irrespective of traditional type 2 biomarker status (Gauvreau et al., 2022).

Tezepelumab, approved by the United States Food and Drug Administration in December 2021, represents the inaugural TSLP-blocking antibody for severe asthma, offering unprecedented therapeutic breadth across phenotypic subtypes (Miralles-López et al., 2024; Hoy, 2022). This review provides an integrative synthesis of tezepelumab’s molecular pharmacology, clinical pharmacokinetic and pharmacodynamic characteristics, pivotal trial evidence, translational biomarker discovery, regulatory positioning, and future research directions.

## 2 Methods

This narrative review synthesizes current evidence on Tezepelumab and TSLP biology in severe asthma. A comprehensive literature search was conducted in PubMed, Scopus, Web of Science, and ClinicalTrials.gov using the following terms: “Tezepelumab,” “thymic stromal lymphopoietin,” “TSLP,” “severe asthma,” “biologic therapy,” and “TSLP blockade.” The search was last updated in November 2025. Peer-reviewed original research articles, clinical trials, systematic reviews, and meta-analyses published in English were included. Case reports and conference abstracts without full-text availability were excluded. Given the heterogeneity of study designs and outcomes, a narrative synthesis approach was employed rather than quantitative meta-analysis.

## 3 Molecular pharmacology of TSLP and Tezepelumab

### 3.1 TSLP biology and receptor signaling

TSLP exists in two alternatively spliced isoforms: the long-form variant (159 amino acids), predominantly upregulated during inflammation by epithelial exposure to proteases, allergens, and viral PAMPs, mediates pro-inflammatory signaling, whereas the short-form constitutive isoform (63 amino acids) contributes to homeostatic immune regulation and epithelial barrier maintenance (Tsilingiri et al., 2017). This isoform switch—from homeostatic to inflammatory TSLP—is a pivotal step in asthma pathogenesis: allergen-activated airway epithelial cells upregulate long-form TSLP expression within hours of exposure, and sputum TSLP mRNA levels correlate significantly with airway eosinophilia, FeNO, and annualized exacerbation frequency in clinical cohorts (Theofani et al., 2023).

TSLP performs its biological role by binding to a high-affinity heteromeric complex composed of the thymic stromal lymphopoietin receptor (TSLPR, also CRLF2) chain and the interleukin-7 receptor alpha chain (IL-7Rα, CD127) (Smolińska et al., 2023). This receptor engagement is structurally asymmetric: TSLP first binds with moderate affinity to TSLPR via its Helix A and the AB loop (Site I), and this binary complex then recruits IL-7Rα via TSLP Helix D (Site II), forming a high-affinity ternary signaling complex. Upon complex formation, TSLPR-associated JAK2 and IL-7Rα-associated JAK1 undergo transphosphorylation. Receptor-associated JAK1 and JAK2 then phosphorylate signal transducer and activator of transcription 5 (STAT5), triggering downstream transcriptional programs (Zhong et al., 2012). Phosphorylated STAT5 homodimerizes, translocates to the nucleus, and drives transcription of OX40 ligand (OX40L) on dendritic cells and cytokine genes IL-5 and IL-13 in ILC2s. In parallel, TSLP receptor engagement activates the PI3K/AKT pathway in airway structural cells and the MAPK/ERK cascade in mast cells, contributing to structural remodeling and histamine release independent of the JAK-STAT axis.

TSLP-activated dendritic cells acquire a distinctive pro-allergic phenotype characterized by robust OX40L expression without IL-12 production, thereby priming naive CD4^+^ T cells toward Th2 differentiation rather than Th1 or regulatory T cell fates (Ebina-Shibuya and Leonard, 2022; Ito et al., 2012). This OX40L-driven Th2 polarization is a master switch for type 2 inflammation: the resulting Th2 cells produce IL-4, IL-5, and IL-13, which drive IgE class switching, eosinophil recruitment, and goblet cell hyperplasia—directly linking the initial TSLP signal to the cardinal features of allergic asthma. Additionally, TSLP directly activates ILC2s, eliciting rapid secretion of IL-5 and IL-13 independent of adaptive immunity, providing a mechanistic explanation for tezepelumab efficacy in patients with minimal eosinophilia (Hoshino et al., 2003). This ILC2-mediated, T cell-independent pathway is particularly relevant in type 2-low patients with recurrent viral exacerbations, where innate immune activation precedes adaptive allergic responses. TSLP also induces migration of airway smooth muscle cells, contributing to remodeling including smooth muscle hyperplasia, subepithelial fibrosis, and mucus hypersecretion (Ji and Li, 2023; Toki et al., 2020). Collectively, this signaling network positions TSLP as a singular upstream node whose activation simultaneously triggers multiple parallel inflammatory programs—providing the mechanistic justification for upstream TSLP blockade as a phenotype-agnostic therapeutic strategy.

### 3.2 TSLP expression and clinical disease severity

A growing body of translational evidence indicates that airway and systemic TSLP levels are higher in asthma and track with indices of disease burden and severity. Bronchial biopsy studies in adults show significantly increased TSLP protein and mRNA expression in airway epithelium and submucosa of asthmatic patients, with the highest levels in those with more severe disease, and TSLP expression correlating inversely with lung function (FEV_1_) and with type 2–related gene signatures (Shikotra et al., 2011). Across multiple specimen types, a recent systematic review and meta-analysis found consistently higher TSLP concentrations in bronchial biopsies and bronchoalveolar lavage fluid (BALF) from asthmatic patients versus healthy controls, and higher blood TSLP in asthma overall, supporting its role as a disease-related marker (Orzołek et al., 2025). In both adults and children, elevated airway or serum TSLP has been linked to worse airflow obstruction, persistent symptoms, and higher exacerbation risk, including in prospective cohorts where higher baseline serum TSLP independently predicted future severe exacerbations and poorer lung function despite high-intensity therapy (Ma et al., 2025; Andreasson et al., 2024). Importantly, TSLP elevation is observed across phenotypes: serum TSLP can be increased in eosinophilic and non-eosinophilic asthma (Ma et al., 2025; Andreasson et al., 2024; Chorvinsky et al., 2022), and sputum or BALF studies show that TSLP associates with airway hyperresponsiveness and inflammation independently of classical type 2 biomarkers (Shrestha et al., 2024). These data provide a mechanistic and biomarker-based rationale for targeting TSLP with tezepelumab across a broad spectrum of severe asthma, including patients who do not exhibit overt type 2–high profiles (Ibrahim et al., 2022).

### 3.3 Tezepelumab’s mechanism of action

Tezepelumab (AMG-157/MEDI9929) is a fully human IgG2λ monoclonal antibody that binds human TSLP with subnanomolar affinity, thereby preventing TSLP from engaging its heterodimeric receptor complex composed of TSLPR and IL-7Rα Nakajima et al., 2020. Biophysical studies show that tezepelumab and related anti-TSLP antibodies bind soluble TSLP and inhibit its interaction with cell-surface TSLPR/IL-7Rα in a concentration-dependent manner, with half-maximal inhibitory concentrations in the low-nanomolar range (Shi et al., 2024). The crystal structure of human TSLP in complex with the tezepelumab Fab fragment, solved at 2.3 Å resolution, reveals that the antibody’s heavy-chain complementarity-determining regions recognize the AB-loop and the C-terminal region of helix D on TSLP, burying ∼1,200 Å^2^ of surface area and forming a polar, hydrogen-bond–rich interface (Verstraete et al., 2017). In this configuration, tezepelumab directly overlaps the TSLPR binding footprint on TSLP while remaining spatially distinct from the IL-7Rα binding site, thereby sterically blocking productive assembly of the TSLP–TSLPR–IL-7Rα signaling complex without occluding the IL-7Rα-facing surface (Bagnasco et al., 2024). These structural data provide a mechanistic explanation for how tezepelumab selectively neutralizes pro-inflammatory TSLP signaling by intercepting the cytokine itself, rather than by targeting either receptor subunit This exquisite structural specificity contributes to the favorable safety profile relative to pan-alarmin approaches (Bagnasco et al., 2024).

By binding and neutralizing TSLP before it engages its receptor complex, tezepelumab inhibits activation of dendritic cells, type 2 innate lymphoid cells, eosinophils, mast cells and other downstream effector cells (Nakajima et al., 2020). In preclinical models, blockade of the TSLP–TSLPR axis attenuates allergen-induced airway eosinophilia, hyperresponsiveness, and structural remodeling, supporting a causal role for TSLP in asthma pathophysiology (Diver et al., 2021).

**FIGURE 1:**
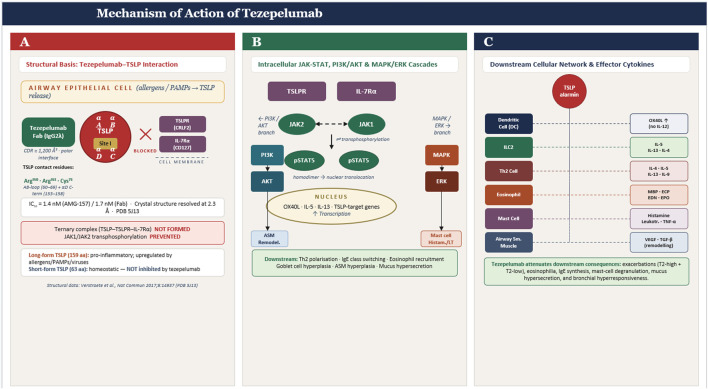
**(A)** TSLP four-helix bundle structure with tezepelumab Fab blocking Site I/TSLPR-binding interface, with key contact residues labeled; **(B)** Intracellular JAK1/JAK2 → pSTAT5 cascade and parallel PI3K/AKT and MAPK/ERK branches with phosphorylation sites; **(C)** Downstream cellular network showing all 6 cell types (DC, ILC2, Th2, eosinophil, mast cell, ASM) with their specific effector cytokines labeled.

The upstream position of TSLP as an epithelial alarmin that coordinates both innate and adaptive immune responses provides a strong mechanistic basis for tezepelumab’s efficacy across diverse asthma endotypes, including eosinophilic and non-eosinophilic disease (Panettieri et al., 2024) (see Figure 1). In contrast to biologics that target single downstream mediators such as IL-5 or IgE, tezepelumab reduces a broad panel of type 2 biomarkers (blood and airway eosinophils, FeNO, IgE, IL-5, IL-13) and improves airway hyperresponsiveness, indicating modulation of multiple inflammatory pathways (Miralles-López et al., 2024).

Clinical trials show that these broad immunologic effects translate into exacerbation reduction across biomarker-defined subgroups, with clinically meaningful benefit even in patients with low type 2 inflammation, although effect sizes are greatest in T2-high disease (Corren et al., 2022). This broader mechanism distinguishes tezepelumab from narrower-spectrum biologics and underlies its therapeutic role in patients with severe asthma, including type 2-low phenotypes that previously lacked targeted options (Menzies-Gow et al., 2021; Sakamoto et al., 2020) (Figure 1).

## 4 Clinical pharmacology and pharmacokinetic/pharmacodynamic profile

### 4.1 Pharmacokinetic characteristics

Tezepelumab exhibits linear, dose-proportional pharmacokinetics following subcutaneous administration across the dose range of 70–280 mg (Table 1) (Sakamoto et al., 2020). The approved dosing regimen of 210 mg subcutaneously every 4 weeks achieves steady-state concentrations within approximately 12 weeks (Zheng et al., 2024). Phase 1 studies in healthy volunteers demonstrated a terminal elimination half-life (t½) of 19.9–25.7 days, consistent with typical IgG pharmacokinetics mediated by neonatal Fc receptor (FcRn) recycling (Parnes et al., 2019). The central and peripheral volumes of distribution are approximately 3.9 L and 2.2 L, respectively, indicating a small overall distribution volume consistent with a monoclonal antibody (Zheng et al., 2024). The absolute subcutaneous bioavailability is ∼77% (Zheng et al., 2024). No clinically meaningful effects of age (≥12 years), sex, race/ethnicity, or renal or hepatic impairment on tezepelumab pharmacokinetics were identified, so dose adjustment is not required for these factors (Zheng et al., 2024; Kurihara et al., 2023). With 210 mg subcutaneously every 4 weeks, the AUC accumulation ratio of ∼1.6–1.8 supports linear, time-invariant pharmacokinetics (Parnes et al., 2019).

**TABLE 1:** Pharmacokinetic parameters of tezepelumab. SC, subcutaneous; Q4W, every 4 weeks; AUC, area under the concentration-time curve.

Parameter	Value	Clinical significance
Dosing regimen	210 mg SC Q4W	Convenient monthly dosing
Terminal half-life	19.9–25.7 days	Supports monthly administration
Time to steady-state	∼12 weeks	Predictable accumulation
The central and peripheral volumes of distribution	3.9 L and 2.2 L	Limited extravascular distribution
Bioavailability (SC)	∼77%	Efficient SC absorption
AUC accumulation ratio	∼1.8	Consistent with linear kinetics
Dose adjustments	None required	No age, sex, race, or organ impairment effects

### 4.2 Pharmacodynamic effects

In the phase 2b PATHWAY trial, tezepelumab 210 mg every 4 weeks produced sustained reductions in key type 2 inflammatory biomarkers over 52 weeks. Compared with placebo, tezepelumab decreased fractional exhaled nitric oxide (FeNO), blood eosinophil counts, and serum total IgE, and lowered multiple downstream mediators including interleukin (IL)-5, IL-13, periostin, thymus and activation-regulated chemokine (TARC), and thymic stromal lymphopoietin (TSLP), indicating broad immunomodulatory effects across the type 2 inflammatory cascade (Corren et al., 2022). Proteomic analyses also showed reductions in matrix-remodelling proteins such as MMP-10 and periostin with tezepelumab versus placebo (Parnes et al., 2019). Pharmacodynamic effects were observed across the full range of baseline type 2 biomarker levels, with reductions seen even in patients with lower blood eosinophil counts (e.g., <150–300 cells/µL) and lower FeNO, although absolute decreases were greatest in those with higher baseline biomarker levels (Emson et al., 2021; Menzies-Gow et al., 2020a).

## 5 Clinical trial evidence

### 5.1 PATHWAY phase 2b trial

The PATHWAY study was a randomized, double-blind, placebo-controlled phase 2b trial that evaluated tezepelumab in adults with uncontrolled moderate-to-severe asthma despite treatment with medium-to-high dose inhaled corticosteroids plus a long-acting β_2_-agonist. In 550 randomized patients, tezepelumab 210 mg subcutaneously every 4 weeks reduced the annualized asthma exacerbation rate (AAER) by 71% versus placebo at 52 weeks (0.19 vs. 0.67 events/patient-year; rate ratio 0.29, 95% CI 0.16–0.51; P < 0.001). Consistent reductions in exacerbations were seen across the full range of baseline blood eosinophil counts, including patients with eosinophil counts <300 cells/µL and even <150 cells/µL, indicating clinically meaningful efficacy in both type 2-high and type 2-low asthma. These findings established proof-of-concept that upstream TSLP blockade with tezepelumab can reduce exacerbations irrespective of traditional type 2 biomarker thresholds and baseline phenotype (Menzies-Gow et al., 2021; Wechsler et al., 2022).

### 5.2 NAVIGATOR phase 3 trial

NAVIGATOR was a phase 3, randomized, double-blind, placebo-controlled trial in 1,061 patients with severe, uncontrolled asthma on medium- or high-dose ICS plus ≥1 controller. Tezepelumab 210 mg Q4W reduced the AAER by 56% versus placebo (0.93 vs. 2.10; rate ratio 0.44, 95% CI 0.37–0.53; P < 0.001) and improved pre-bronchodilator FEV_1_ by 0.13 L over placebo at week 52 (0.23 vs. 0.09 L; P < 0.001). Benefits in exacerbations, lung function, asthma control and HRQoL were consistent across baseline eosinophil strata, including BEC <300 cells/µL (Nordenmark et al., 2023).

### 5.3 SOURCE phase 3 OCS-sparing trial

The SOURCE trial was a randomized, double-blind, placebo-controlled phase 3 study that evaluated the oral corticosteroid (OCS)–sparing effect of tezepelumab in 150 adults with severe, OCS-dependent asthma. The primary endpoint — a categorised reduction in daily maintenance OCS dose at week 48 — was not met in the overall population (odds ratio [OR] 1.28, 95% CI 0.69–2.35; P = 0.43). However, in the pre-specified subgroup of patients with baseline blood eosinophil counts ≥150 cells/μL, tezepelumab was associated with significantly greater OCS dose reduction compared with placebo (OR 2.58, 95% CI 1.16–5.75). Tezepelumab was well tolerated across the study population, with no new safety signals identified (Wechsler et al., 2022).

### 5.4 CASCADE airway remodeling study

The phase 2 CASCADE trial evaluated tezepelumab’s effects on airway inflammation and structure in adults with moderate-to-severe uncontrolled asthma using bronchoscopic biopsies and exploratory CT-based airway imaging. Tezepelumab produced a marked reduction in airway submucosal eosinophils versus placebo (geometric least-squares mean ratio 0.15, 95% CI 0.05–0.41; nominal p < 0.001) across baseline biomarker subgroups. Although no significant changes were seen in reticular basement membrane thickness or epithelial integrity over ∼28 weeks, CT analyses from CASCADE showed reduced mucus plug scores and associated improvements in airway lumen size and lung function with tezepelumab compared with placebo. These reductions in airway eosinophilia, mucus plugging, and downstream type 2 biomarkers provide mechanistic support for effects that extend beyond symptomatic control alone, consistent with potential disease-modifying activity (Lipworth et al., 2025a; Pelaia et al., 2021).

## 6 Safety and tolerability

Tezepelumab has demonstrated robust efficacy and a favorable safety profile across multiple clinical trials in severe asthma. The Phase 2b PATHWAY and Phase 3 NAVIGATOR studies showed substantial reductions in annualized asthma exacerbation rates (up to ∼71%) and improvements in lung function across a broad range of baseline blood eosinophil counts, including type 2-low populations (Menzies-Gow et al., 2021; Edris et al., 2019). The CASCADE mechanistic study provided evidence of reduced airway inflammation and remodeling, with significant decreases in submucosal eosinophils, airway smooth muscle mass, and inflammatory biomarkers, supporting potential disease-modifying effects beyond symptom control. The SOURCE trial demonstrated clinically meaningful oral corticosteroid–sparing effects in patients dependent on maintenance OCS, with higher odds of ≥50% dose reduction and greater rates of OCS discontinuation versus placebo. Pooled Phase 2/3 safety data and the long-term DESTINATION extension confirmed that tezepelumab is generally well tolerated, with adverse event rates comparable to placebo, low and non-neutralizing anti-drug antibody development, and no new safety signals over up to 3 years of exposure (Menzies-Gow et al., 2021; Edris et al., 2019).

## 7 Discussion

Tezepelumab represents a conceptual and therapeutic advance in severe asthma because it targets the upstream epithelial alarmin TSLP, which initiates and sustains multiple inflammatory pathways, rather than single downstream effector cytokines such as IL-5, IL-4/13, or IgE. Acting at the top of the inflammatory cascade allows broader modulation of airway inflammation, with reductions in diverse type 2 biomarkers (blood and airway eosinophils, FeNO, IgE, IL-5, IL-13) and effects on both eosinophilic and non-eosinophilic mechanisms (Kyriakopoulos et al., 2024). Across randomized trials and real-world studies, tezepelumab consistently reduces exacerbations and improves lung function, symptoms, and quality of life regardless of baseline eosinophils or FeNO, including in type 2-low and non-T2 phenotypes where other biologics are often ineffective. Network meta-analysis and comparative reviews highlight its particularly strong exacerbation reduction across biomarker strata and suggest advantages over some downstream biologics in eosinophil-low subgroups. This broad, phenotype-spanning efficacy expands options for patients who remain uncontrolled on maximal inhaled therapy or have failed other biologics limited to allergic or eosinophilic asthma, supporting tezepelumab as a first-in-class, upstream strategy that can address multiple disease pathways and evolving phenotypes over time (Pitre et al., 2022; Lugogo et al., 2025).

### 7.1 Mechanistic comparison with other approved asthma biologics

A rigorous mechanistic comparison requires analysis at the level of target molecule, position in the inflammatory hierarchy, specific signaling branches inhibited, cell types affected, and resulting phenotypic applicability. The fundamental distinction between tezepelumab and all other approved asthma biologics is its upstream positioning: current biologics such as mepolizumab, benralizumab, dupilumab, and omalizumab target individual downstream mediators (IL-5/IL-5Rα, IL-4/13Rα, or IgE) within the type 2 cascade, whereas tezepelumab blocks the epithelial alarmin TSLP, which initiates and sustains multiple innate and adaptive inflammatory pathways in response to allergens, viruses, bacteria, and pollutants (Menzies-Gow et al., 2020b).

Mepolizumab and benralizumab selectively inhibit the IL-5 axis, leading to eosinophil depletion and reduced eosinophilic airway inflammation, but they do not directly interfere with TSLP-driven dendritic cell activation, ILC2 stimulation, or broader epithelial alarmin signaling and are therefore mainly effective in eosinophilic/type 2-high phenotypes, with limited benefit in type 2-low or paucigranulocytic asthma. Dupilumab blocks IL-4Rα and thereby IL-4/IL-13 signaling, attenuating STAT6-dependent type 2 responses (e.g., IgE synthesis, mucus production, type 2 airway inflammation), but it still operates downstream of epithelial alarmins and does not directly suppress all TSLP-initiated innate pathways (Menzies-Gow et al., 2021).

Tezepelumab, by binding TSLP and preventing its interaction with the TSLP receptor complex, exerts broad downstream effects, with reductions in blood and airway eosinophils, FeNO, IgE, IL-5, IL-13, and other biomarkers, and has demonstrated efficacy across allergic, eosinophilic, and non-eosinophilic phenotypes, including patients with low blood eosinophils and low FeNO. In the phase 3 NAVIGATOR trial, tezepelumab significantly reduced exacerbations and improved lung function irrespective of baseline eosinophil count, including patients with BEC <300 cells/µL, supporting clinically meaningful activity beyond classic eosinophilic disease. Mechanistic biopsy data from CASCADE further show reductions in airway submucosal eosinophils and improved airway hyperresponsiveness without major effects on other inflammatory cell populations, consistent with targeted yet upstream modulation of TSLP-driven pathways.

Despite its upstream position, long-term clinical trials report a safety profile comparable to placebo (Table 2) with no signal of broad immunosuppression, suggesting that tezepelumab’s structural specificity for TSLP–TSLPR interactions translates into selective inhibition of TSLP-initiated signaling while largely sparing homeostatic immunity (Emson et al., 2021; Pelaia et al., 2021; Menzies-Gow et al., 2020b).

**TABLE 2:** Safety profile of tezepelumab (pooled data from Phase 2/3 trials).

Adverse event	Tezepelumab (%)	Placebo (%)
Any adverse event	76	79
Nasopharyngitis	21	22
Upper respiratory tract infection	12	13
Headache	10	9
Injection-site reactions	4	3
Serious adverse events	10	13
Anaphylaxis	<1	<1
Helminth infections	<1	<1
Anti-drug antibodies	5–7	—
Discontinuation due to AE	3	4

Network meta-analyses have provided valuable insights in the absence of head-to-head trials (Shrestha et al., 2024). A Bayesian network meta-analysis (Nopsopon et al., 2023) demonstrated that tezepelumab was associated with significantly lower exacerbation rates than benralizumab (relative risk 0.63, 95% credible interval 0.46–0.86), but FEV_1_ comparisons with mepolizumab and dupilumab yielded overlapping credible intervals, precluding conclusions of superiority (Park et al., 2022). Tezepelumab, mepolizumab, and dupilumab each achieved >99% probability of reducing exacerbation rates by ≥ 50% versus placebo, while benralizumab achieved only 66% probability (Park et al., 2022). The absence of prospective head-to-head trials remains a critical evidence gap; indirect comparisons are inherently limited by between-trial heterogeneity and should be interpreted with caution (Table 3).

**TABLE 3:** Systematic mechanistic comparison of approved biologics for severe asthma (revised: 6 columns, MOA and signaling pathway columns added).

Biologic	Target	Hierarchy	Signaling inhibited	Phenotypic breadth	Key limitation
Tezepelumab	TSLP (long-form)	Upstream alarmin	JAK1/JAK2/STAT5; PI3K/MAPK; ILC2, DC, mast, ASM	T2-high AND T2-low (BEC <150: 48%–50% AAER ↓)	Attenuated benefit in double-negative T2-low
Mepolizumab	IL-5	Downstream cytokine	IL-5R/JAK2/STAT5; eosinophil survival	T2-high only (BEC ≥150 cells/µL)	No T2-low benefit; no DC/ILC2 blockade
Benralizumab	IL-5Rα	Downstream receptor	IL-5R/JAK2; ADCC eosinophil depletion	T2-high only (BEC ≥150 cells/µL)	No T2-low benefit; rapid depletion without upstream inhibition
Dupilumab	IL-4Rα	Downstream shared receptor	STAT6; IgE class switching; mucus	T2-high preferred; partial T2-low benefit	No ILC2/mast cell/neutrophilic pathway blockade
Omalizumab	Free IgE	Downstream effector	IgE-FcεRI; mast cell/basophil degranulation	Allergic T2-high only	IgE eligibility ceiling; no non-allergic benefit

BEC, blood eosinophil count; AAER, annualized asthma exacerbation rate; DC, dendritic cell; ASM, airway smooth muscle.

Regarding pharmacoeconomic positioning, tezepelumab carries a significant acquisition cost (∼$28,000–$35,000 per year in the US). ICER has suggested a health-benefit price benchmark of $9,000–$12,100 per year, and CADTH reported an incremental cost-effectiveness ratio exceeding $1,300,000 per QALY gained (Rind et al., 2022; Canadian Agency for Drugs and Technologies in Health (CADTH), 2023). Cost-effectiveness analyses in Taiwan and Brazil demonstrated more favorable profiles when accounting for exacerbation-related hospitalizations and corticosteroid burden reduction (Rind et al., 2022; Canadian Agency for Drugs and Technologies in Health (CADTH), 2023). As real-world evidence accumulates, pharmacoeconomic models are expected to be revised to capture the full value of upstream phenotype-agnostic TSLP blockade (Pavord et al., 2025).

### 7.2 Predictive biomarkers and patient selection

Optimal patient selection for tezepelumab therapy represents a critical unresolved question (Park et al., 2022). Although tezepelumab’s broad efficacy is advantageous, identifying candidates with the highest likelihood of substantial benefit versus modest improvement remains challenging in the absence of validated predictive biomarkers (Park et al., 2022). Post-hoc analyses from NAVIGATOR suggest that patients with higher baseline exacerbation rates, elevated FeNO levels, and allergic comorbidities may derive greater absolute benefit. In allergic asthma, pooled analyses demonstrate a 62% exacerbation reduction (95% CI: 53%–70%) in patients with perennial aeroallergen sensitization, with an 80% reduction in exacerbations requiring hospitalization. In eosinophilic asthma (BEC ≥300 cells/µL), tezepelumab achieves 71%–73% exacerbation reductions, comparable or superior to anti-IL-5/IL-5R biologics in network meta-analyses. Emerging evidence suggests multi-omics composite signatures and AI/machine learning integration may identify optimal tezepelumab responders (Pitre et al., 2022).

### 7.3 Limitations of current evidence

Critical appraisal of tezepelumab’s evidence base reveals several important limitations. First, clinical trial populations were selected using stringent criteria that may not reflect real-world severe asthma heterogeneity, including elderly individuals and those with multiple comorbidities. Second, the 52-week duration of pivotal trials provides limited insight into long-term disease modification and durability of response after discontinuation, though randomized extension studies up to approximately 2 years provide reassurance on sustained efficacy and safety while exploratory post-cessation follow-up is ongoing (Lugogo et al., 2025). Third, heterogeneity of trial designs and populations across biologic comparators complicates indirect treatment comparisons, despite adjustment strategies in network meta-analyses (Menzies-Gow et al., 2020b).

### 7.4 Treatable traits framework

The treatable traits model conceptualizes asthma as overlapping pulmonary, extrapulmonary and behavioral traits, rather than a single disease label, and targets each trait with specific interventions. Severe asthma registries show patients typically express multiple traits simultaneously (median ≈10 traits/person, including eosinophilic inflammation, frequent exacerbations, allergic sensitization, upper airway disease and psychological comorbidity). These supports describing severe asthma as a syndrome of overlapping pathobiological processes, comorbidities and environmental/behavioral factors (Priessnitz et al., 2025; Biene et al., 2024).

### 7.5 Airway remodeling and disease modification

In the phase 2 CASCADE trial, tezepelumab significantly reduced airway submucosal eosinophils versus placebo and improved airway hyperresponsiveness to mannitol, while reticular basement membrane thickness and epithelial integrity (key airway remodelling measures) did not change significantly, indicating a predominant effect on airway inflammation rather than demonstrable reductions in airway smooth muscle mass (Pavord et al., 2025). Computed-tomography analyses from CASCADE further showed that tezepelumab reduced occlusive mucus plug scores and increased airway lumen size, with these changes correlating with improvements in lung function, supporting potential disease-modifying effects on airway obstruction beyond symptomatic control.

A pooled *post hoc* analysis of PATHWAY and NAVIGATOR reported that among patients with abnormal pre-bronchodilator lung function at baseline, 17.2% of tezepelumab recipients versus 9.9% of placebo recipients achieved normal lung function (pre-bronchodilator FEV_1_ ≥80% predicted; ≥90% for adolescents) at week 52, indicating that tezepelumab can restore normal spirometry in a meaningful subset of patients with severe, uncontrolled asthma (Menzies-Gow et al., 2023).

Long-term extension data from DESTINATION show that with continued dosing for up to 104 weeks (≈2 years total exposure), tezepelumab maintains a favorable safety profile and sustains clinically meaningful reductions in exacerbations, alongside improved lung function, asthma control, and health-related quality of life. Extended off-treatment follow-up indicates that biomarker suppression (blood eosinophils, FeNO, IgE) and clinical benefits (FEV_1_, asthma control) gradually wane after cessation and return toward placebo levels without fully reverting to baseline over 36–40 weeks, underscoring the importance of ongoing therapy and highlighting that the durability of benefit after discontinuation and any long-term disease-modifying effects off treatment remain key knowledge gaps (Menzies-Gow et al., 2020b).

## 8 Future directions

### 8.1 Expansion into non-asthma indications

TSLP is a key epithelial “alarmin” implicated in allergic rhinitis, atopic dermatitis, eosinophilic esophagitis, CRSwNP, and COPD, providing a strong mechanistic basis for evaluating tezepelumab beyond asthma. In CRSwNP, TSLP (including highly active cleaved forms) is elevated in polyps and non-polyp mucosa and drives type 2 inflammation, which is dose-dependently inhibited *ex vivo* by tezepelumab. A phase 3 trial (WAYPOINT) showed that tezepelumab significantly reduced nasal-polyp size, nasal congestion, sinonasal symptoms, and the need for surgery and systemic glucocorticoids compared with placebo in adults with severe CRSwNP. In atopic dermatitis, a phase 2a trial found numerical but not statistically significant improvements in EASI responses when tezepelumab was added to topical corticosteroids, indicating modest benefit so far. Tezepelumab is also under clinical development or has orphan designation for eosinophilic esophagitis, COPD, and chronic spontaneous urticaria, but phase 3 efficacy data are not yet available (Hoy, 2022; Lipworth et al., 2025b; Priessnitz et al., 2025).

### 8.2 Real-world evidence generation

Since approval, real-world studies have broadened understanding of tezepelumab’s effectiveness beyond trial populations, including patients under-represented or excluded from PATHWAY/NAVIGATOR. The phase 4 PASSAGE study enrolled a heterogeneous US cohort (Black/African American patients, adolescents, current/former smokers, and patients with comorbid mild–moderate COPD) and showed a 76% reduction in annualized asthma exacerbation rate (AAER) overall, with comparator reductions in smokers (71%) and COPD–asthma overlap (66%), and clinically meaningful improvements in lung function and asthma control, confirming that trial efficacy extends to these groups. European and other multicenter cohorts from Germany, Italy and Spain similarly report large AAER reductions (≈67–86%), improved ACT scores and oral corticosteroid sparing, without loss of effect in low-eosinophil or T2-low disease. Across these registries, 39%–63% of patients had prior biologic exposure; switching from anti-IL-5/5R or other mAbs to tezepelumab still yielded substantial exacerbation reductions and improved control, although responses are somewhat attenuated compared with biologic-naive patients (Lugogo et al., 2025; Biene et al., 2024; Gates et al., 2025).

Clinical remission has now been quantified in large real-world cohorts: in a 175-patient United Kingdom series, 36% achieved clinical remission at 1 year, with higher rates in T2-high (55%) than T2-low phenotypes (19%), and similar remission proportions in biologic-naive and biologic-experienced patients. However, only 38% achieved “biological remission” (normalization of blood eosinophils and FeNO), and just 15% met both clinical and biological remission criteria, demonstrating a consistent disconnect between symptom control and complete suppression of type 2 inflammation, whose long-term implications remain uncertain (Gates et al., 2025).

### 8.3 Clinical remission as a treatment goal

The notion of clinical remission in severe asthma has emerged as an aspirational treatment target that extends beyond mere reduction of exacerbations or day-to-day symptom control. In a large real-world cohort of adults with severe uncontrolled asthma treated with tezepelumab, on-treatment clinical remission at 12 months—defined by absence of exacerbations and oral corticosteroid use, good symptom control, and preserved lung function—was achieved in 36% of patients overall (Gates et al., 2025), with substantially higher rates in those with a type 2 (T2)-high inflammatory profile (55%) compared with T2-low patients (19%). Notably, only a minority of clinical remitters also fulfilled criteria for biological remission (normalization of blood eosinophils and fractional exhaled nitric oxide), highlighting a disconnect between clinical and biological remission, the long-term prognostic implications of which remain uncertain and require further study (Table 4) (Gates et al., 2025).

**TABLE 4:** Real-world evidence for tezepelumab: key studies and subgroup findings (new table, added in revision).

Study	Design/N	Key subgroups	Effectiveness outcome	Prior biologic exposure
PASSAGE (Lugogo et al., 2025)	Phase 4, open-label, US, N = 280 (interim)	Black/AA ≥30%; adolescents; COPD-overlap; smokers	ACT improvement consistent across all subgroups; AAER −51%–56%	Biologic-naive and experienced
Poto et al., 2025	Multicenter RWE, Italy, N = 211	T2-high vs. T2-low; prior anti-IL-5 exposure	ACT +5.1 pts; AAER −63% naive vs. −58% experienced	38% prior anti-IL-5/IL-5Rα failure
Biene et al., 2024	Multicenter RWE, Germany, N = 226	Allergic (68.2%) vs. eosinophilic (31.8%) phenotype	No significant difference in AAER reduction by phenotype	Prior biologic use ∼40%
Gates et al., 2025	RWE, United Kingdom, N = 94	T2-high vs. T2-low; clinical vs. biological remission	Clinical remission: 36% overall; 55% T2-high; 19% T2-low at 12 months	Mixed (biologic-naive and experienced)

ACT, Asthma Control Test; AAER, annualized asthma exacerbation rate; AA, African American.

### 8.4 Next-generation TSLP-Targeted therapeutics

Emerging next-generation approaches are exploring dual and multi-target inhibition of epithelial alarmins and downstream pathways. These include bispecific antibodies simultaneously neutralizing TSLP and IL-33 or combining anti-TSLP with other inflammatory targets (for example, stem cell factor or IL-4Rα), as well as engineered TSLP “traps” and other biologics or small-molecule strategies that disrupt TSLP receptor signaling (Matera et al., 2020). Preclinical data suggest that concurrent blockade of multiple alarmins can more effectively suppress type 2–driven airway inflammation than single-cytokine inhibition, supporting the hypothesis that multi-alarmin or multi-pathway targeting may yield synergistic efficacy in patients with complex, refractory disease where monotherapy proves insufficient (Matera et al., 2020; Yu et al., 2025).

## 9 Conclusion

Tezepelumab constitutes a first-in-class advance in severe asthma therapy by targeting thymic stromal lymphopoietin (TSLP), an epithelial alarmin situated at the apex of multiple inflammatory networks, thereby exerting broad effects across phenotypes independent of conventional type 2 (T2) biomarker status 38. By binding TSLP at its receptor-interaction site, tezepelumab prevents activation of downstream pathways involving type 2 cytokines (IL-4, IL-5, IL-13), IgE, eosinophils, mast cells, group 2 innate lymphoid cells, and structural airway cells, leading to reductions in key inflammatory biomarkers, airway hyperresponsiveness, and exacerbation risk.

Across phase 2b and 3 trials, tezepelumab reduced annualized asthma exacerbation rate by approximately 56%–71% versus placebo, with clinically meaningful benefits observed in eosinophilic, allergic, mixed allergic–eosinophilic, T2-low, and oral-corticosteroid-dependent phenotypes. Indirect comparisons suggest numerically lower exacerbation rates with tezepelumab relative to several other approved biologics, though without statistically significant superiority in formal network analyses 18. Its pharmacokinetic profile supports fixed 210-mg subcutaneous dosing every 4 weeks, and safety data from large randomized trials and real-world cohorts indicate tolerability comparable to placebo, without new safety signals.

Real-world studies corroborate trial findings, demonstrating improved symptom control, lung function, biomarker reduction, and oral corticosteroid sparing across both T2-high and T2-low populations, including patients previously exposed to other biologics. Exploratory bronchoscopy data show reductions in airway eosinophils and hyperresponsiveness, but consistent effects on structural remodeling indices have not yet been established (Pavord et al., 2025; Lipworth et al., 2025b; Caminati et al., 2023).

Key outstanding needs include validated predictors of response, direct head-to-head trials versus other biologics, and long-term data on airway remodeling and durable disease modification.
